# ALDH/CD44 identifies uniquely tumorigenic cancer stem cells in salivary gland mucoepidermoid carcinomas

**DOI:** 10.18632/oncotarget.5782

**Published:** 2015-09-22

**Authors:** April Adams, Kristy Warner, Alexander T. Pearson, Zhaocheng Zhang, Hong Sun Kim, Daiki Mochizuki, Gregory Basura, Joseph Helman, Andrea Mantesso, Rogério M. Castilho, Max S. Wicha, Jacques E. Nör

**Affiliations:** ^1^ Department of Restorative Sciences, University of Michigan School of Dentistry, Ann Arbor, Michigan, USA; ^2^ Department of Internal Medicine, University of Michigan Medical Center, Ann Arbor, Michigan, USA; ^3^ Department of Otolaryngology, University of Michigan School of Medicine, Ann Arbor, Michigan, USA; ^4^ Department of Oral and Maxillofacial Surgery, University of Michigan School of Medicine, Ann Arbor, Michigan, USA; ^5^ Department of Oral Pathology, University of Sao Paulo School of Dentistry, São Paulo, Brazil, USA; ^6^ Department Periodontics Oral Medicine, University of Michigan School of Dentistry, Ann Arbor, Michigan, USA; ^7^ Department of Biomedical Engineering, University of Michigan College of Engineering, Ann Arbor, Michigan, USA; ^8^ Comprehensive Cancer Center, University of Michigan, Ann Arbor, Michigan, USA

**Keywords:** salivary gland cancer, tumorigenesis, self-renewal, multipotency, tumor initiating cells

## Abstract

A small sub-population of cells characterized by increased tumorigenic potential, ability to self-renew and to differentiate into cells that make up the tumor bulk, has been characterized in some (but not all) tumor types. These unique cells, namedcancer stem cells, are considered drivers of tumor progression in these tumors. The purpose of this work is to understand if cancer stem cells play a functional role in the tumorigenesis of salivary gland mucoepidermoid carcinomas. Here, we investigated the expression of putative cancer stem cell markers (ALDH, CD10, CD24, CD44) in primary human mucoepidermoid carcinomas by immunofluorescence, *in vitro* salisphere assays, and *in vivo* tumorigenicity assays in immunodeficient mice. Human mucoepidermoid carcinoma cells (UM-HMC-1, UM-HMC-3A, UM-HMC-3B) sorted for high levels of ALDH activity and CD44 expression (ALDH^high^CD44^high^) consistently formed primary and secondary salispheres *in vitro*, and showed enhanced tumorigenic potential *in vivo* (defined as time to tumor palpability, tumor growth after palpability), when compared to ALDH^low^CD44^low^ cells. Cells sorted for CD10/CD24, and CD10/CD44 showed varying trends of salisphere formation, but consistently low *in vivo* tumorigenic potential. And finally, cells sorted for CD44/CD24 showed inconsistent results in salisphere formation and tumorigenic potential assays when different cell lines were evaluated. Collectively, these data demonstrate that salivary gland mucoepidermoid carcinomas contain a small population of cancer stem cells with enhanced tumorigenic potential and that are characterized by high ALDH activity and CD44 expression. These results suggest that patients with mucoepidermoid carcinoma might benefit from therapies that ablate these highly tumorigenic cells.

## INTRODUCTION

Advanced salivary gland mucoepidermoid carcinoma (MEC) is a relentless and tyspically fatal disease. Mucoepidermoid carcinoma is the most common malignant salivary gland cancer, accounting for 5-15% of all salivary tumors and 30-35% of malignant salivary tumors [1-7]. These tumors arise in both the major and minor salivary gland and are characterized by the presence of mucous, epidermoid, and intermediate cells types. Low-grade tumors show noticeable cyst formation, a higher portion of mucous cells, and minimal cytological mutation while high-grade tumors are characterized by large concentrations of intermediate and squamous cells as well as increased mitotic activity. Current treatment consists of surgical resection with or without radiation, depending on tumor grade. Patients presenting with recurrent, locally invasive, or metastatic tumors do not have effective treatment options [8]. Understanding the pathobiology of this cancer, particularly mechanisms involved in resistance to therapy, is critical to improve the survival and the quality of life of patients with mucoepidermoid carcinoma.

The cancer stem cell (CSC) hypothesis states that tumors contain a small sub-population of multipotent cells that are capable of self-renewal and differentiation, and are uniquely tumorigenic. These cells initiate and maintain tumor growth and progression in several cancers including breast, head and neck, pancreatic, liver, ovarian, colorectal, and brain cancers [9–16]. However, it is unclear if cancer stem cells play a functional role in the pathobiology of mucoepidermoid carcinoma. Importantly, cancer stem cells are thought to be resistant to chemotherapy and radiation due, at least in part, to slower proliferation rates and differential function of transporter proteins [17–19]. It is believed that survival of these cells after treatment enables tumor relapse. Identification and understanding of how these cells function in mucoepidermoid carcinomas might lead to more effective therapies.

Isolation of cancer stem cells can be accomplished using protein markers that are differentially expressed in stem cells compared to the non-cancer stem cell population. One such marker is aldehyde dehydrogenase (ALDH)-1, a cytosolic enzyme that oxidizes aldehydes into carboxylic acids [20–22]. ALDH1 is thought to play an important role in hematopoietic stem cell fate determination by regulating the conversion of retinol into retinoic acid [23]. Importantly, ALDH1 identifies cancer stem cells in breast, lung, head and neck, colorectal, ovarian, pancreatic, bladder, prostate, and cervical cancers [10, 24–33]. Another surface marker protein used extensively to identify cancer stem cells is CD44, a transmembrane glycoprotein. This protein functions in key cellular processes regulating survival, differentiation, growth, and cell motility [34]. CD44 has been used as a stem cell marker in breast, head and neck, pancreatic, prostate, and colorectal cancers [9, 34]. The cell adhesion protein CD24 is also an important stem cell marker used in breast, pancreatic, and colorectal cancers [9, 35, 36]. Interestingly, cancer stem cells are identified within the CD24^low^ population in breast tumors [9], while in pancreatic cancer they are identified within the CD24^high^ population [35]. And finally, the metallo-endoprotease CD10, a diagnostic marker in several tumors, has been implicated in invasion is breast, gastric, and colorectal cancer. This protein plays an important role in the maintenance of mammary gland stem cells, suggesting that it could also serve as a marker for stem cells in glandular malignancies [37].

While cancer stem cells have been identified and well characterized in several tumors, their presence and functional role has not been investigated in salivary gland mucoepidermoid carcinomas. Here, we used cell lines and xenograft models recently generated in our laboratory [38] to screen for cancer stem cells using several combinations of ALDH, CD44, CD24, and CD10 markers. Our findings indicate that cancer stem cells play a functional role in mucoepidermoid carcinoma, and that these cells can be isolated using the ALDH/CD44 marker combination. In contrast, combinations of CD44, CD24, and CD10 did not identify uniquely tumorigenic cells consistently. Together, these results unveil the function of a uniquely tumorigenic population of cancer stem cells in the pathogenesis of mucoepidermoid carcinomas.

## RESULTS

### Characterization of putative stem cell markers in human mucoepidermoid carcinomas

**Figure 1:**
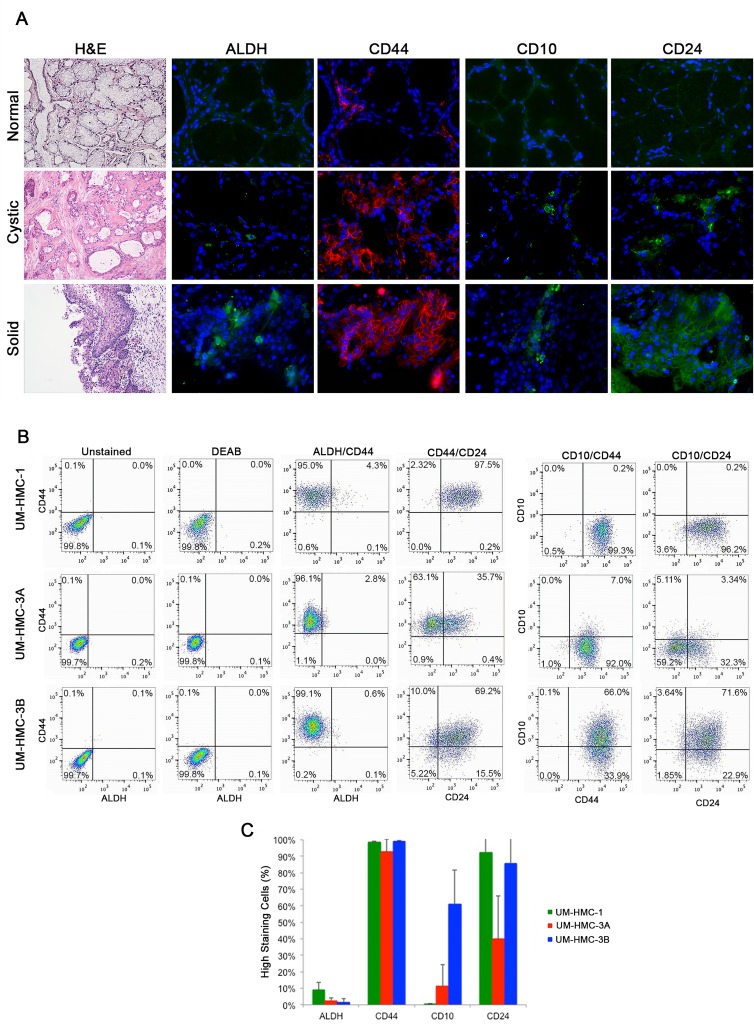
Characterization of putative stem cell markers in human mucoepidermoid carcinoma specimens **A.** Representative photomicrographs of H&E and immunofluorescence images of tissue sections derived from patients with normal salivary gland; a low-grade, cystic tumor; or a high-grade, solid tumor. ALDH1, CD10, and CD24 are stained in green while CD44 is stained in red. H&E images were taken at 40X and immunofluorescence images were taken at 400X. **B.** Flow cytometry analysis of three cell lines (UM-HMC-1, UM-HMC-3A, UM-HMC-3B) stained for ALDH/CD44, CD44/CD24, CD10/CD44, and CD10/CD24. CD44-APC staining is shown on the horizontal axis while ALDH staining is shown on the vertical axis. CD44-PE is shown on the horizontal axis and CD10-APC is in the vertical axis. CD24-FITC is shown on the horizontal axis and CD10-APC or CD44-APC is shown on the vertical axis. **C.** Graph depicting the percentage of positive cells for ALDH, CD44, CD10, and CD24.

To investigate the expression patterns of cancer stem cell markers in human mucoepidermoid carcinomas, we obtained tissue sections from diagnostic incisional biopsies and performed immunofluorescence staining. We focused on stem cell markers that have been verified in other glandular malignancies, *i.e.* ALDH, CD44, CD24, and CD10. We found that 7 of the 12 samples showed positive staining for all four markers. Ten of 12 samples stained positively for ALDH1, 12 of 12 samples stained for CD44, 9 of 12 samples stained for CD10, and 10 of the 12 samples stained for CD24 (Table 1). Interestingly, we observed low staining levels for each one of these markers in normal salivary glands, when qualitatively compared with mucoepidermoid carcinomas (Figure 1A).

When less aggressive, cystic tumors were compared to more aggressive, solid tumors, we saw an increase in ALDH1 expression in the solid tumor (Figure 1A). In contrast, CD44 stained highly in both tumor types (Figure 1A). CD10 and CD24 showed differential expression between the cystic and solid tumor types. CD10 showed expression in both the cystic and solid tumors, however, more positive staining was seen in the solid tumor (Figure 1A). Interestingly, cells with high CD10 expression were localized mainly on the outside edge near the stroma suggesting that these cells may be important in intercellular signaling with the microenvironment. Tumor cells in these sections showed positive staining for CD24. However, the solid tumor areas showed more positive staining when compared to the cystic areas (Figure 1A). Together, these results suggest that ALDH1, CD44, CD10, and CD24 are highly expressed in salivary gland mucoepidermoid carcinoma when compared to normal salivary gland and that expression of ALDH1, CD10, and CD24 may be differentially regulated in more aggressive cell types.

We also performed immunofluorescence staining on three human salivary mucoepidermoid carcinoma cell lines (UM-HMC-1, UM-HMC-3A, UM-HMC-3B) plated in Lab-Tek glass slides. We observed that ALDH1 staining is present but in only few cells (Supplementary Figure S1). In contrast, CD44 stained very highly in all cell lines evaluated (Supplementary Figure S1). CD10 stained positively but its expression was variable among the cell lines (Supplementary Figure S1). While UM-HMC-3B stained highly for CD10, UM-HMC-1 showed significantly less CD10 expression. UM-HMC-3A showed moderate staining when compared to UM-HMC-1 and UM-HMC-3B. Finally, all three cell lines showed similar levels of expression of CD24.

**Table 1:** Patient demographic and expression of CSC markers in human salivary gland mucoepidermoid carcinomas

Case Number	Gender	Age (Years)	Localization	H&E Predominant Morphology	Tentative Grading	Tumor Size (mm)	Immunofluorescence Staining			
							ALDH	CD44	CD10	CD24
1	F	49	Hard palate	Mixed	Intermediate	10	Absent	Present	Absent	Present
2	M	46	Jugal mucosa	Solid	High	8	Present	Present	Absent	Present
3	F	24	Hard palate	Solid	Intermediate	30	Present	Present	Present	Present
4	F	14	Hard/soft palate	Solid	High	50	Present	Present	Present	Present
5	F	29	Palate	Mixed	High	20	Present	Present	Present	Present
6	F	26	Palate	Cystic	Low	15	Present	Present	Present	Present
7	F	NA	Hard palate	Cystic	Low	6	Absent	Present	Present	Absent
8	F	46	Retromolar region/vestibule	Solid	High	40	Present	Present	Present	Present
9	M	62	Jugal mucosa	Cystic	High	12	Present	Present	Absent	Present
10	F	63	Hard palate	Solid	Intermediate	15	Present	Present	Present	Absent
11	F	67	Palate	Solid	Intermediate	15	Present	Present	Present	Present
12	F	55	Hard palate	Mixed	High	20	Present	Present	Present	Present

### Characterization of putative stem cell markers in mucoepidermoid carcinoma cell lines

We used flow cytometry to screen three human salivary mucoepidermoid carcinoma cell lines (UM-HMC-1, UM-HMC-3A, UM-HMC-3B) for putative cancer stem cell markers. UM-HMC cells consistently showed greater than 90% positive staining for CD44. In contrast, the percentage of ALDH high cells in UM-HMC-1 was only 4.4%, and in UM-HMC-3A and UM-HMC-3B was even lower (2.8%, 0.7% respectively) (Figure 1B and 1C). When these two markers were combined, the most common population of cells was consistently ALDH^low^CD44^high^ (Figure 1B).

UM-HMC cells showed variable staining for CD10 and CD24. UM-HMC-1 and UM-HMC-3B stained highly for CD24, while UM-HMC-3A showed less staining (Figure 1B and 1C). CD10 staining was highest in UM-HMC-3B when compared to UM-HMC-3A and UM-HMC-1 cells (Figure 1C). The combination of CD10/CD24 in UM-HMC-1 showed the majority of cells as CD10^low^CD24^high^. In UM-HMC3A cells, most cells stained CD10^low^CD24^low^ followed by CD10^low^CD24^high^, CD10^high^CD24^low^, and CD10^high^CD24^high^. UM-HMC-3B cells stained highly for CD10^high^CD24^high^ cells followed by CD10^low^CD24^high^, CD10^high^CD24^low^, and CD10^low^CD24^low^, showing an inverse expression profile when compared with UM-HMC-3A (Figure 1B). When stained for combination CD44/CD24, UM-HMC-3B and UM-HMC-1 stained predominately CD44^high^CD24^high^, while UM-HMC-3A stained mainly CD44^high^CD24^low^. While all cell lines stained positively for CD44, CD24 expression was variable (Figure 1B and 1C). CD10/CD44 combination also showed differential expression among cell lines. UM-HMC-1 and UM-HMC-3A showed low staining for CD10 and therefore, the most prevalent population in both lines was CD10^low^CD44^high^. UM-HMC-3B staining positively for CD10^high^CD44^high^ but also showed staining in the CD10^low^CD44^high^ population (Figure 1B).

In summary, all UM-HMC cell lines showed positive staining for the four markers studied here. We observed that all cell lines consistently presented low ALDH activity and high CD44 expression. On the other hand, the expression of CD10 and CD24 was highly variable from cell line to cell line.

### *In vitro* salisphere analysis of mucoepidermoid carcinoma cell lines

To begin the functional characterization of these putative marker combinations, we screened the UM-HMC cell lines for salisphere formation under ultra-low attachment, serum-free conditions. The three cell lines studied here formed salispheres. However, UM-HMC-1 cells generated less salispheres than UM-HMC-3A and UM-HMC-3B under these culture conditions (Supplementary Figure S2A and S2B). To evaluate the effectiveness of each specific marker combination to select cells with enhanced self-renewal capacity, primary salispheres were dissociated and passaged into secondary salispheres (Supplementary Figure S2C). Interestingly, we observed a trend for increasing number of salispheres with passaging when unsorted cells were evaluated (Supplementary Figure S2A).

**Figure 2:**
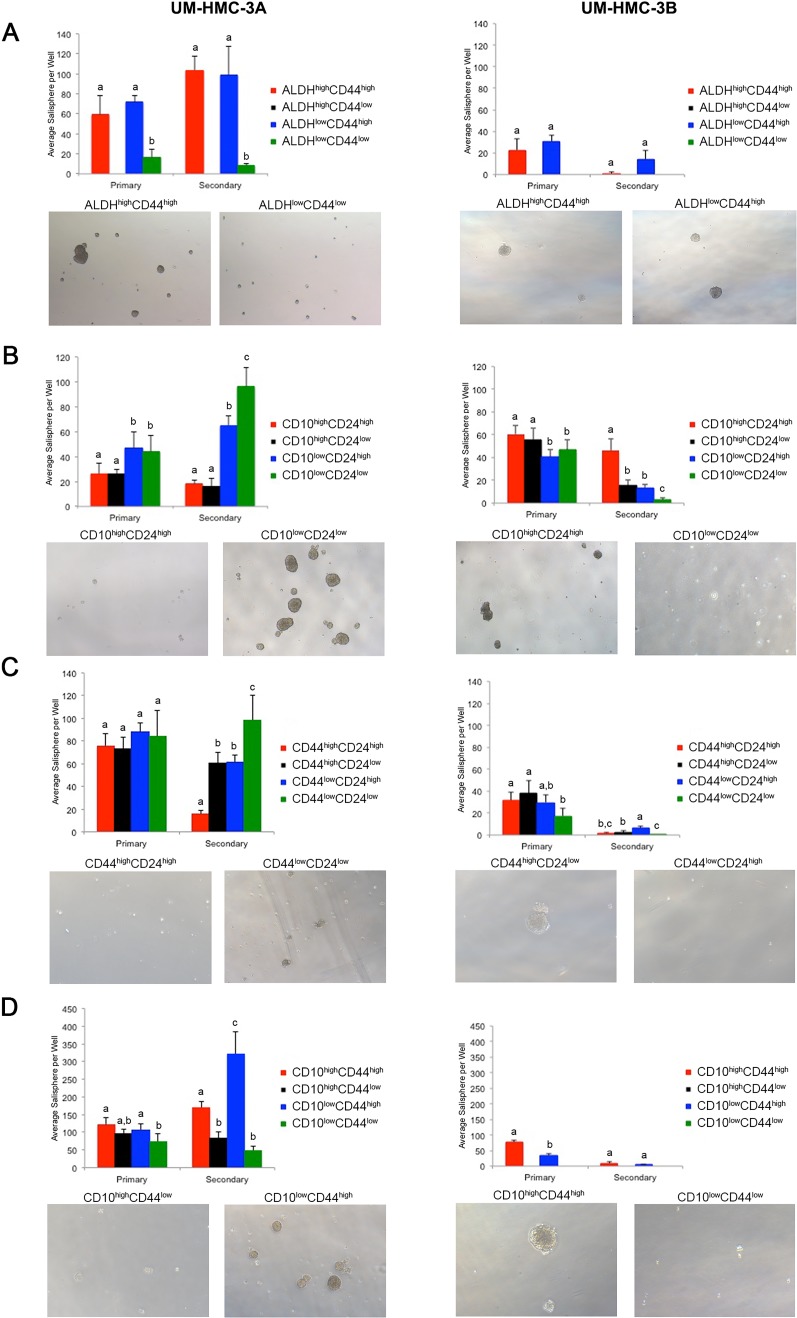
*In vitro* salisphere analysis of FACS-sorted mucoepidermoid carcinoma cell lines (UM-HMC-3A, UM-HMC-3B) **A.–D.** Ultra-low attachment plates were seeded with 2,000 cells/well (6-well plates), and cells were cultured for seven days to generate primary salispheres. Then, salispheres were dissociated into single cell suspensions, seeded in new ultra-low attachment plates, and secondary salispheres were counted after additional seven days. **A.** Graph depicting the average number of salispheres per well of cell lines FACS-sorted for ALDH/CD44 (*n* = 4-6). **B.** Graph depicting the average number of salispheres per well of cell lines FACS-sorted for CD10/CD24 expression (*n* = 5-6). **C.** Graph depicting the average number of salispheres per well of cell lines FACS-sorted for CD44/CD24 cells (*n* = 6). D, Graph depicting the average number of salispheres per well of cell lines FACS-sorted for CD10/CD44 sorted cells (*n* = 5-6). All images were taken at 40X. Statistical analysis was performed using one-way ANOVA. Different low case letters indicate statistical difference at *p* < 0.05.

To begin to understand the ability of marker combinations to select for cancer stem cells, we FACS-sorted the UM-HMC-3A and UM-HMC-3B cell lines according to ALDH activity, CD10, CD24, and/or CD44 protein expression. Sorted cells were plated in ultra-low attachment conditions and grown for seven days before the number of salispheres was determined. Salispheres were then dissociated and allowed to grow for additional seven days under the same culture conditions. The ALDH^low^CD44^low^ cells showed little to no salisphere growth. In contrast, both the ALDH^high^CD44^high^ and ALDH^low^CD44^high^ populations showed significant salisphere formation in primary and secondary cultures (Figure 2A, Table 2). Because the ALDH^high^CD44^low^ population is so rare, we were unable to obtain sufficient cell numbers to be analyzed.

Cells sorted for CD10/CD24 showed significant differences in the number of salispheres. In the UM-HMC-3A cells, the CD10^low^CD24^low^ population significantly outgrew the other populations. The CD10^low^CD24^high^ population also showed considerable salisphere formation in this cell line. Interestingly, the UM-HMC-3B cells showed an outgrowth of the CD10^high^CD24^high^ population in secondary salispheres (Figure 2B, Table 2). UM-HMC-3A cells sorted according to CD44/CD24 marker combination also showed significant differences in salisphere formation, specifically in the CD44^low^CD24^low^ population. In contrast, UM-HMC-3B cells showed growth in the CD44^low^CD24^high^ population in secondary salispheres (Figure 2C, Table 2). Finally, UM-HMC-3A and UM-HMC-3B cells were sorted by CD10/CD44. In the UM-HMC-3A cells, the CD10^low^CD44^high^ population formed the most secondary salispheres. In the UM-HMC-3B cells, the only populations that had sufficient numbers to enable us to perform this assay were the CD10^high^CD44^high^ and CD10^low^CD44^high^ cells. We observed that CD10^high^CD44^high^ formed significantly more primary salispheres than the CD10^low^CD44^high^ cells (Figure 2D, Table 2).

We observed that the marker combinations tested here showed different patterns of salisphere growth. ALDH^high^CD44^high^ and ALDH^low^CD44^high^ populations showed consistent salisphere formation, and therefore this combination was selected for the first *in vivo* studies (see below). The CD10/CD24, CD44/CD24, and CD10/CD44 marker combinations showed significant variability in salisphere growth. Nevertheless, these marker combinations were also tested *in vivo* for tumorigenic potential.

**Table 2:** *In vitro* salisphere formation and in vivo tumorigenic potential of cells selected by the following putative CSC marker combinations

ALDH/CD44	Salisphere Formation		*In Vivo* Tumorigenicity	
	UM-HMC-3A	UM-HMC-3B	Low-Passage	High-Passage
High/High	High	High	High	High
High/Low	NA	NA	NA	NA
Low/High	High	High	NA	NA
Low/Low	Low	Low	Low	Low
CD10/CD44				
			
High/High	Low	High	NA	Low
High/Low	Low	Intermediate	NA	Low
Low/High	Intermediate	Intermediate	NA	None
Low/Low	High	Low	NA	None
CD44/CD24				
			
High/High	Low	Low	Low	High
High/Low	Intermediate	Low	Low	Low
Low/High	Intermediate	High	High	Low
Low/Low	High	Low	Low	Intermediate
CD10/CD44				
			
High/High	Intermediate	High	None	NA
High/Low	Low	NA	NA	NA
Low/High	High	Intermediate	NA	NA
Low/Low	Low	NA	Low	NA

### Combination of ALDH activity and CD44 expression selects highly tumorigenic cells

**Figure 3:**
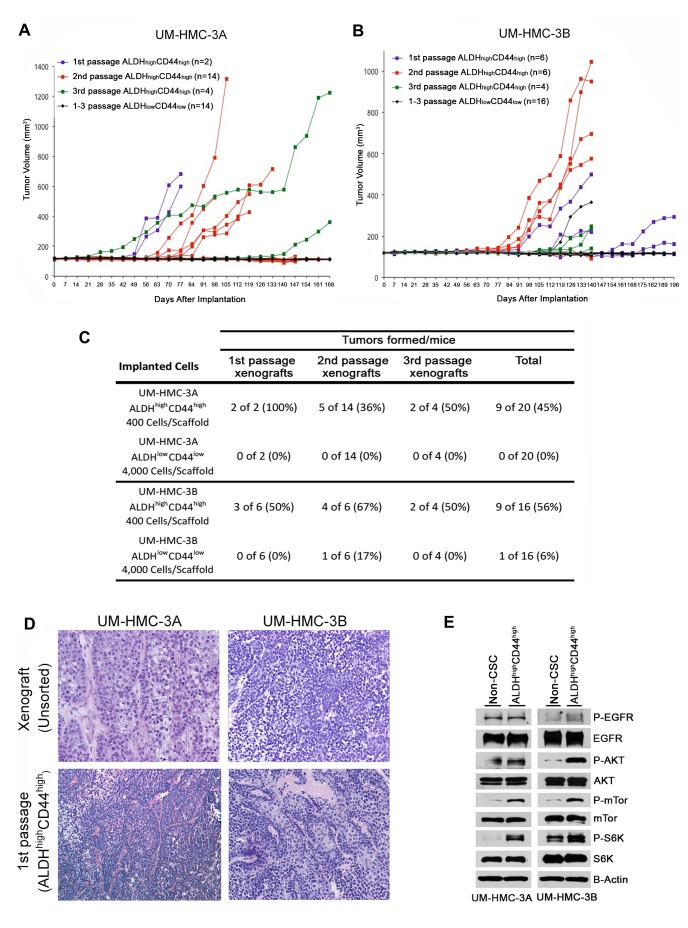
Tumorigenic potential of low passage mucoepidermoid carcinoma cells sorted for ALDH/CD44 **A., B.** Graphs depicting tumor volume of **A.** UM-HMC-3A or **B.** UM-HMC-3B xenograft cells FACS-sorted for ALDH/CD44. Scaffolds were seeded with either 400 ALDH^high^CD44^high^ or 4,000 ALDH^low^CD44^low^ cells and transplanted into the subcutaneous space of SCID mice. Existing tumors were retrieved, re-sorted and 400 ALDH^high^CD44^high^ or 4,000 ALDH^low^CD44^low^ cells seeded into new scaffolds, and serially passaged *in vivo*. **C.** Table depicting the number of tumors grown in the ALDH^high^CD44^high^ *versus* ALDH^low^CD44^low^ populations for each passage performed. **D.** H&E staining of tumors generated with FACS-sorted ALDH^high^CD44^high^ and ALDH^low^CD44^low^ cells. Images were taken at 100X. **E.** UM-HMC-3A and UM-HMC-3B cells were sorted for ALDH^high^CD44^high^ or combined ALDH^high^CD44^low^, ALDH^low^CD44^high^, and ALDH^low^CD44^low^ (non-CSC population). NP-40 lysis buffer was used to prepare whole cell lysates that were resolved using PAGE. Membranes were probed using antibodies a 1:1000 dilution against human mTor, p-mTor, Akt, p-Akt, S6K, p-S6K, p-EGFR; 1:2000 dilution of EGFR, and beta-actin.

As a critical follow-up to the *in vitro* studies, putative cancer stem cell markers were verified *in vivo* to ascertain self-renewal and tumorigenic potential. We first decided to FACS-sort for ALDH/CD44 and implant these cells *in vivo* to observe possible differences in tumorigenic potential. Because of the extended length of time needed to grow low passage cell line-derived tumors, we digested UM-HMC-3A (passage 14) and UM-HMC-3B (passage 27) xenograft tumors and then sorted these cells for ALDH/CD44. The sorted cells were seeded with primary human endothelial cells into biodegradable scaffolds, and transplanted into the SCID mice, as we showed [38–40]. Either 400 of the ALDH^high^CD44^high^ cells, or 4,000 of the ALDH^low^CD44^low^ cells (*i.e.* 10x more cells), were transplanted into mice and serially passaged *in vivo*. In the first generation xenografts, we observed that only ALDH^high^CD44^high^ cells generated tumors (Figure 3A and 3B). Interestingly, ALDH^high^CD44^high^-sorted cells were able to generate tumors with similar histology as compared to the tumors generated from the unsorted cells (Figure 3D). We next took the tumors generated with ALDH^high^CD44^high^ cells, digested, stained, re-sorted, and transplanted 400 ALDH^high^CD44^high^ or 4,000 ALDH^low^CD44^low^ cells into new mice. While the ALDH^high^CD44^high^ cells generated tumors in 9/20 transplants, ALDH^low^CD44^low^ cells generated tumors in only 1/20 transplants (Figure 3A and 3B). Finally, we did a third cycle of *in vivo* passaging of the ALDH^high^CD44^high^ tumors. Here, only mice transplanted with ALDH^high^CD44^high^ cells generated tumors (Figure 3A and 3B). Notably, no secondary tumors were generated from the only ALDH^low^CD44^low^ tumor that grew in this experiment. Overall, we observed 18 tumors generated with 400 ALDH^high^CD44^high^ cells, while only one tumor was generated when 4,000 ALDH^low^CD44^low^ cells were transplanted (Figure 3C). Collectively, these data showed that ALDH^high^CD44^high^ cells exhibit enhanced tumorigenic potential, when compared with ALDH^low^CD44^low^ cells. Notably, the unique tumorigenic potential of ALDH^high^CD44^high^ cells persisted over multiple *in vivo* tumor passages, suggesting enhanced self-renewal of this sub-population of cells.

As the ALDH^high^CD44^high^ showed elevated tumorigenic potential, we performed western blot analysis to see if UM-HMC-3A and UM-HMC-3B ALDH^high^CD44^high^ cells showed activation of the PI2K-Akt pathway important in cancer stem cells function. While the levels of EGFR and phosphor-EGFR remained stable between the combined ALDH^high^CD44^low^, ALDH^low^CD44^high^, and ALDH^low^CD44^low^ populations (non-stem cell) and ALDH^high^CD44^high^ cells, there was an upregulation of phosphor-mTor and phospho-S6K in the UM-HMC-3A cells (Figure 3E). In the UM-HMC-3B cells, we also observed an upregulation of p-mTor and p-S6K as well as an upregulation of p-Akt (Figure 3E). Together these results suggest that the PI3K-Akt pathway is upregulated in the ALDH^high^CD44^high^ compared to the non-stem cell population.

**Figure 4:**
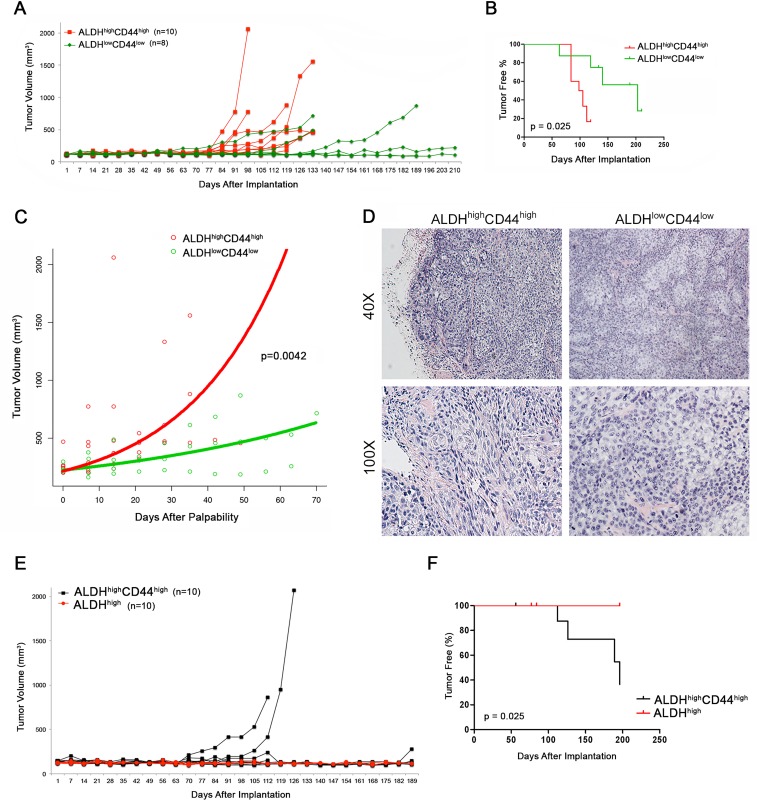
Tumorigenic potential of high passage mucoepidermoid carcinoma cells sorted for ALDH/CD44 **A.** Graph depicting the volume of tumors generated by the transplantation of FACS-sorted UM-HMC-3B cells (ALDH^high^C44^high^ or ALDH^low^CD44^low^) in immunodeficient mice. 5,000 sorted UM-HMC-3B cells (passage 103) and 900,000 endothelial (HDMEC) cells were seeded on biodegradable scaffolds and transplanted into the subcutaneous space of SCID mice. Tumors were measured weekly and mice were euthanized once the tumors reached 700-1,500 mm^3^. **B.** Kaplan-Meyer analysis of time to palpability of tumors generated with ALDH^high^CD44^high^ or ALDH^low^CD44^low^ cells. Tumors were considered palpable once they reached 200 mm^3^. **C.** Regression analysis of growth after palpability (200 mm^3^) of tumors generated with FACS-sorted ALDH^high^CD44^high^ or ALDH^low^CD44^low^ cells. **D.** H&E staining of tumors generated with FACS-sorted ALDH^high^CD44^high^ and ALDH^low^CD44^low^ cells. Images were taken at 40X and 100X. **E.** Graph depicting the volume of tumors generated by the transplantation of FACS-sorted UM-HMC-3B cells (ALDH^high^C44^high^ or ALDH^high^) in immunodeficient mice. 5,000 sorted UM-HMC-3B cells (passage 104) and 900,000 endothelial (HDMEC) cells were seeded on biodegradable scaffolds and transplanted into the subcutaneous space of SCID mice. Tumors were measured weekly and mice were euthanized once the tumors reached 700-1,500 mm^3^. **F.** Kaplan-Meyer analysis of time to palpability of tumors generated with ALDH^high^C44^high^ or ALDH^high^ cells.

We next wanted to understand whether these differences in tumorigenic potential where reproducible using higher passage cells in independent *in vivo* experiments. We sorted UM-HMC-3B cells (passage 103) for ALDH^high^CD44^high^ and ALDH^low^CD44^low^, seeded the sorted cells with primary human endothelial cells into biodegradable scaffolds, and transplanted them into the SCID mice. Tumors were measured weekly and considered palpable once they reached 200 mm^3^ (Figure 4A, Table 2). Kaplan-Meyer analysis demonstrated that the tumorigenic potential of ALDH^high^CD44^high^ cells was higher than the ALDH^low^CD44^low^ cells (log-rank test, *p* = 0.025) (Figure 4B). We performed regression analysis to determine the impact of ALDH/CD44 marker combination on tumor growth rate. Once tumors had grown to 200 mm^3^ we performed a linear mixed effect model on the tumor size, including the following variables in our model of log tumor volume: size of tumor at first palpability; ALDH^high^CD44^high^ state; time; time by ALDH^high^CD44^high^ cell state interaction (Figure 4C). As expected, the volume of the tumor increased proportionally to the size of the initially palpable tumor (*p* = 0.0094), as well as with time (*p* = 0.0037). There was also a significant increase in tumor growth rate for ALDH^high^CD44^high^ tumors compared to ALDH^low^CD44^low^ tumors (*p* = 0.0042). We plotted the time since first-palpability *versus* tumor volume. Overlaid on this graph are the model-derived growth predictions. To generate the curves for each group, we used the mean size at time of first palpability for each group, and the appropriate estimated coefficients and interactions from the model. Tumors generated with ALDH^high^CD44^high^ cells showed a distinctly different morphology from the tumors generated with ALDH^low^CD44^low^ cells (Figure 4D). Both are characterized by large solid areas, but tumors generated with ALDH^high^CD44^high^ cells showed more intermediate-like cells, with spindle shape, oval nuclei and highly anaplastic areas. In contrast, the tumors generated with ALDH^low^CD44^low^ cells showed a more monotonous morphology with round cells exhibiting round nuclei and clusters of epidermoid-like cells with eosinophilic cytoplasm. Interestingly, anaplastic cells were more rare in the tumors generated with ALDH^low^CD44^low^ cells.

As the majority of high passage UM-HMC-3B cells stain highly for CD44, we next questioned whether ALDH could be used as a single marker for this aggressive cancer stem cell phenotype. To investigate this, we took high passage (passage 104) UM-HMC-3B cells and sorted for ALDH^high^CD44^high^ and ALDH^high^ then transplanted these cells with human endothelial cells on biodegradable scaffolds into the SCID mice. In these studies, we were able to generate tumors in 4 (out of 10) scaffolds seeded with ALDH^high^CD44^high^ cells while no tumors were generated in the ALDH^high^ cells (Figure 4E). Our Kaplan-Meyer analysis shows that the tumorigenic potential of ALDH^high^CD44^high^ cells is greater than the ALDH^high^ cells (log-rank test, p-0.025) (Figure 4F). These data suggest that ALDH by itself does not enrich for an aggressive cancer stem cell phenotype in salivary gland mucoepidermoid carcinoma.

**Figure 5:**
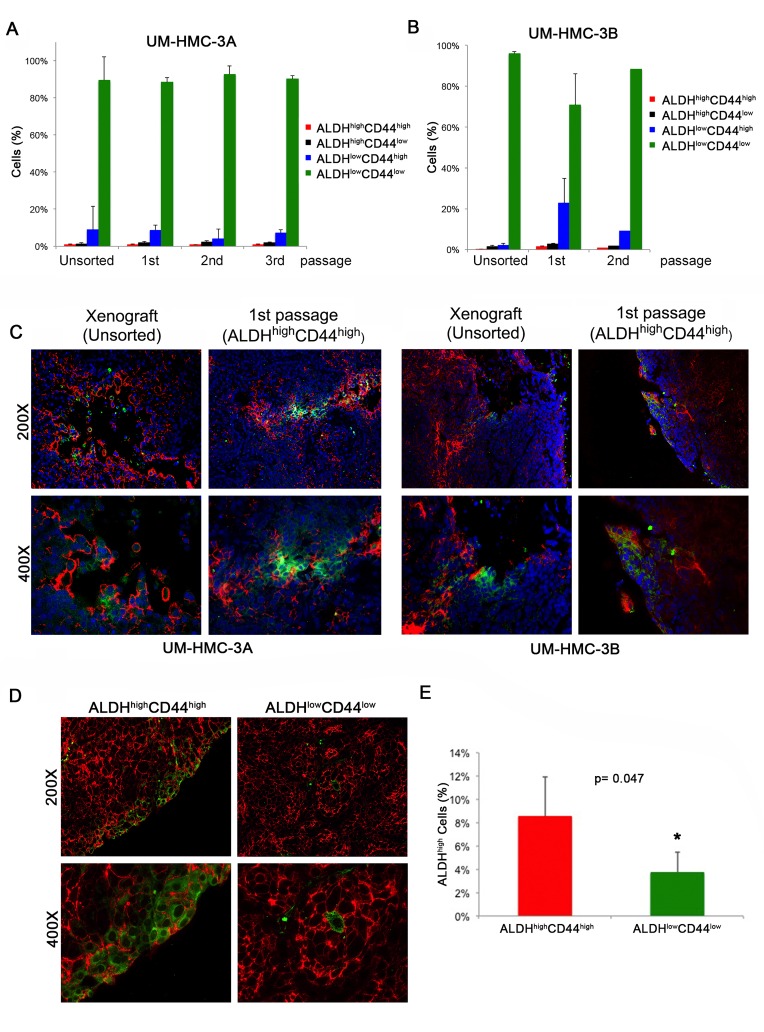
Characterization of xenograft tumors generated with cells sorted for ALDH/CD44 **A.** Graph depicting the percentage of ALDH/CD44 cells in tumors generated with FACS-sorted UM-HMC-3A (passage 14) over three *in vivo* passages. **B.** Graph depicting the percentage of ALDH/CD44 staining cells in tumors generated with FACS-sorted UM-HMC-3B (passage 27) over two *in vivo* passages. **C.** Immunofluorescence staining of tumors generated from the original unsorted UM-HMC-3A (passage 14) and UM-HMC-3B (passage 27) xenograft cells compared to tumors generated from 400 ALDH^high^CD44^high^ cells sorted from the original xenografts. CD44 is stained in red while ALDH-1 is stained in green. **D.** Immunofluorescence staining of tumors generated with FACS-sorted ALDH^high^CD44^high^ or ALDH^low^CD44^low^ cells. CD44 is stained in red while ALDH-1 is stained in green. **E.** Graph depicting the percentage of ALDH^high^ cells in tumors generated with cells FACS-sorted for ALDH^high^CD44^high^ or ALDH^low^CD44^low^ as determined by flow cytometry with Aldefluor.

We next performed FACS analysis of the ALDH/CD44 sorted xenograft tumors over multiple passages to verify if ALDH^high^CD44^high^ cells were able to differentiate. We observed that tumors generated with pure populations of ALDH^high^CD44^high^ cells were able to continuously repopulate the other ALDH/CD44 sub-populations and that the fraction of the different sub-populations was consistent with the original unsorted xenograft tumors (Figure 5A and 5B). We also performed immunofluorescence staining of the original unsorted tumors and compared with the 1^st^ passage ALDH^high^CD44^high^-sorted tumors. We found once again that the ALDH^high^CD44^high^ generated tumors were able to repopulate the remaining three sub-populations (Figure 5C). We also performed immunofluorescence staining in tumors generated from our second independent experiment with FACS-sorted ALDH^high^CD44^high^ cells or ALDH^low^CD44^low^ cells to determine the ability of these relatively pure sub-populations of cells to regenerate complex tumors once transplanted in mice. We found that CD44 stained ubiquitously the vast majority of the cells in all tumors, including those generated with ALDH^low^CD44^low^ cells. The pattern of ALDH expression was different. Tumors generated with FACS-sorted ALDH^high^CD44^high^ showed more ALDH1 staining than tumors generated with ALDH^low^CD44^low^ cells (Figure 5D and 5E). Interestingly, the presence of cells that are positive for the stem cell marker ALDH1 in tumors generated with FACS-sorted ALDH^low^CD44^low^ cells suggests that perhaps some of these cells are capable of dedifferentiation. Nevertheless, the percentage of ALDH^high^ cells was lower in the tumors generated with ALDH^low^CD44^low^ cells when compared to tumors generated with ALDH^high^CD44^high^ cells (Figure 5E).

### Tumorigenic potential of mucoepidermoid carcinoma cells FACS-sorted for CD10/CD24, CD44/CD24, CD10/CD44

In addition to the work performed with ALDH/CD44, we have also performed extensive testing of three additional putative stem cell marker combinations (CD10/CD24, CD44/CD24, CD10/CD44) to determine if these markers could enrich for cancer stem cells *in vivo*. We transplanted UM-HMC-3B FACS-sorted cells (CD10/CD24 or CD10/CD44) into mice, as described above. We observed only two tumors generated upon transplantation of the CD10/CD24-sorted cells, 1 (out of 6) in the CD10^high^CD24^high^ group and 1 (out of 6) in the CD10^high^CD24^low^ group (Supplementary Figure S3A, Table 2). Further, only two CD10^low^CD44^low^ tumors were formed when CD10/CD44-sorted cells were transplanted (Supplementary Figure S3B, Table 2). These data demonstrated that these two marker combinations involving CD10 do not select for uniquely tumorigenic cancer stem cells.

**Figure 6:**
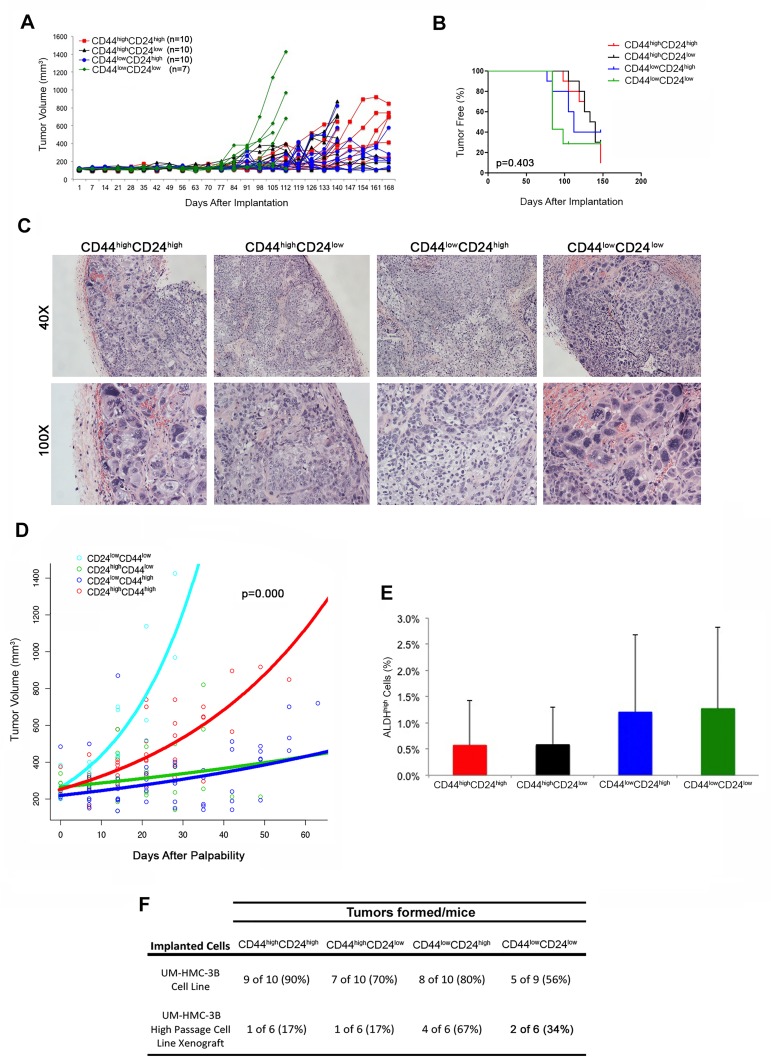
Tumorigenic potential of mucoepidermoid carcinoma cells sorted for CD44/CD24 **A.** *In vivo* transplantation of 5,000 UM-HMC-3B (passage 103) FACS-sorted cells (CD44^high^CD24^high^, CD44^high^CD24^low^, CD44^low^CD24^high^, or CD44^low^CD24^low^) with 900,000 endothelial (HDMEC) cells seeded on biodegradable scaffolds and transplanted into the subcutaneous space of SCID mice. **B.** Kaplan-Meyer analysis of time to palpability of tumors generated with cell sorted for CD44/CD24. Tumors were considered palpable once they reached 200 mm^3^. **C.** H&E staining of tumors generated by the transplantation of UM-HMC-3B cells sorted for CD44/CD24. Images were taken at 40X and 100X. **D.** Regression analysis of growth after palpability (200 mm^3^) of tumors generated with cells FACS-sorted for CD44 and CD24. **E.** Graph depicting the percentage of ALDH^high^ cells in tumors generated with cells FACS-sorted for CD44/CD24. **F.** Table depicting the number of tumors formed in each CD44/CD24 sorted sup-population in both the UM-HMC-3B cell line and the UM-HMC-3B low passage cell line xenograft model.

We next performed experiments with the CD44/CD24 marker combination using cells that were sorted from ongoing UM-HMC-3B xenograft tumors. Because different sub-populations of CD44/CD24 are used to isolate cancer stem cells in different cancer types, we FACS-sorted all four sub-populations and implanted them as described above. Four (out of 6) CD44^low^CD24^high^ transplants grew tumors, whereas only 1 (out of 6) mouse transplanted with CD44^high^CD24^high^ or CD44^high^CD24^low^, and 2 (out of 6) mice developed tumors when transplanted with CD44^low^CD24^low^ cells (Supplementary Figure S3C; Figure 6F, Table 2). While these initial results suggested that CD44^low^CD24^high^ sub-populations were more tumorigenic, we did not observe significant differences in the time to palpability upon Kaplan-Meyer analyses (Supplementary Figure S3D), nor did we observe that a certain sub-population of cells generated tumors with a particularly aggressive histology (Supplementary Figure S3E). We then digested the tumors and re-stained for CD44/CD24 to analyze how the sub-populations of cells redistributed during growth in mouse. In the original xenograft tumors used to collect the cells for this experiment, the CD44^low^CD24^low^ sub-population was the lowest (Supplementary S3F). In contrast, in the tumors generated from FACS-sorted cells, we observed an overall trend for high fractions of CD44^low^CD24^low^ cells irrespective of what was the sub-population used to generate the tumors.

These surprising results led us to repeat this experiment using cells sorted directly from the UM-HMC-3B cell line. In this experiment, the four sub-populations of cells sorted for CD44/CD24 were able to grow tumors (Figure 6A, 6F, Table 2), but no difference was seen in the time to palpability (*p* = 0.403) among these four experimental conditions (Figure 6B). Nevertheless, we did observe significant differences in tissue morphology. The CD44^low^CD24^low^ and CD44^high^CD24^high^ tumors grew more aggressively and showed a solid morphology with large areas of anaplasia. In contrast, CD44^high^CD24^low^ and CD44^low^CD24^high^ cells generated tumors exhibiting a less aggressive, mucous cell phenotype (Figure 6C). We again performed regression analyses to determine if differences in tumor growth rates existed based on the different sub-populations of cells that were used to generate these tumors. Based on the significant histologic differences that we observed between four combinations of CD24 and CD44 cells, we included the following variables in our model of log tumor volume: size of tumor at first palpability; CD24 state; CD44 state; CD24 by time interaction; CD44 by time interaction; and CD24 by CD44 by time interaction. The rate of tumor growth was significantly less in CD24-positive (*p* = 0.0003), and CD44-positive (*p* = 0.0003) tumors compared to the negative populations. There was a significant interaction effect, which yielded a higher rate of growth for CD44^high^CD24^high^ tumors (*p* < 0.0001). We again plotted the time since first-palpability *versus* tumor volume (Figure 6D). To further investigate the absence of differences in tumor initiating potential (as determined by time to palpability) among the cells sorted for CD44/CD24, we analyzed whether any sub-population was enriched for ALDH. Interestingly, no significant difference in the fraction of ALDH^high^ cells was observed when we compared tumors generated with FACS-sorted CD44^high^CD24^high^, CD44^high^CD24^low^, CD44^low^CD24^high^, or CD44^low^CD24^low^ cells (Figure 6E). Collectively, these data indicate that the CD44/CD24 marker combination does not enable consistent identification of a unique population of highly tumorigenic cells in salivary gland mucoepidermoid carcinomas.

## DISCUSSION

Poor survival of patients with advanced stage salivary gland mucoepidermoid carcinomas demand better understanding of the pathobiology of these tumors and the development of new, mechanism-based therapies. Research in other cancer types suggests that cancer stem cells play an important role in resistance to therapy and tumor relapse [18, 41–44]. Much has been done to therapeutically to target the self-renewal pathways important in cancer stem cell function. Several groups have therapies to inhibit the Notch, Wnt, and Hedgehog pathways [45]. In addition, Her2 specific antibodies have been used to target breast cancer stem cells while IL-6 antibodies have been used in head and neck squamous cell carcinomas [46, 27]. The relentless growth of mucoepidermoid carcinomas, compounded with resistance to every therapy that was attempted this far, is a major clinical challenge that might be correlated with the function of cancer stem cells. However, whether or not cancer stem cells play a role in the pathobiology of salivary mucoepidermoid carcinomas has not been investigated due a lack of adequate research models (*i.e.* cell lines, xenograft models) and unavailability of markers that enable the identification of sub-populations of cells with unique tumorigenic potential. Previously, we generated and characterized a number of cells lines and xenograft models of salivary gland mucoepidermoid carcinoma [38].

By co-transplanting sorted human mucoepidermoid carcinoma cells with primary human microvascular endothelial cells in biodegradable scaffolds, we were able to generate xenograft tumors vascularized with human blood vessels, as previously described [38–40]. We have showed that this experimental approach enables the crosstalk between tumor cells and endothelial cells of the same species, which has a demonstrable impact to both tumor growth as well as response to therapy [47]. Here, we demonstrated that the combination of ALDH activity and CD44 expression enables the identification of highly tumorigenic cells in salivary gland mucoepidermoid carcinoma. While primary cells are a preferred model to study, we are limited by the rarity by these tumors, as well as the difficulty and length of time needed to grow primary cells *in vitro* and *in vivo*. However, the results presented here, together with the recent characterization of cell lines and xenograft models of mucoepidermoid carcinoma [38], will enable studies focused on the understanding of the mechanisms underlying the role of cancer stem cells in resistance to therapy, and the development of strategies to overcome this resistance.

While the salisphere assay is a useful method to screen for cancer stem cells markers *in-vitro*, the ability of these markers to enrich for cells that are able to self-renew and are multipotent must be verified *in-vivo*. Most of these *in vivo* experiments lasted around 200 days, and some of them lasted more than one year (*e.g.* sequential *in vivo* passaging of sorted cells). The extended time necessary to achieve tumor palpability, and the relatively slow tumor growth after palpability consumed significant resources and delayed the progression of this work. However, we believe that the results observed in these preclinical experiments reflect the normal behavior of human mucoepidermoid carcinomas, which are slow growing, albeit relentless, tumors.

A series of complementary independent *in vivo* studies demonstrated that the ALDH/CD44 marker combination enriches for cancer stem cells in mucoepidermoid carcinomas. While ALDH can be used as an independent marker for cancer stem cells in other cancer types, we have demonstrated that a two-marker combination of ALDH and CD44 is necessary to enrich for this aggressive cancer stem cell phenotype. In contrast, cells sorted for CD10/CD24 or CD10/CD44 showed differences in salisphere formation, but poor ability to generate tumors *in vivo*. We concluded that these marker combinations do not enrich for cancer stem cells, at least in the models studied here. We also concluded that the CD44/CD24 combination does not enrich for cancer stem cells. In this case, we observed differences in salisphere formation and tumor growth. However, the sub-populations of interest were not consistent from experiment to experiment. Interestingly, several tumors generated in these experiments were very aggressive, showing solid morphology with large areas of anaplasia. Paradoxically, these aggressive tumors were observed primarily when cells sorted for CD44^high^CD24^high^ or CD44^low^CD24^low^ were transplanted. The mechanistic understanding of these puzzling findings is beyond the scope of this manuscript. However, these data reinforced the concept that the CD44/CD24 combination is likely not a viable marker combination for mucoepidermoid carcinoma cancer stem cells.

The PI3K-Akt signaling pathway has been found to be important in the maintenance of cancer stem cells [48, 49]. Interestingly, the EGFR and HER2-Akt-mTOR pathways are activated in salivary gland cancer [50]. We observed that ALDH^high^CD44^high^ cells potently express P-mTor and p-S6K, when compared to control cells. Considering the promising results of clinical and preclinical studies with rapamycin and rapalogs, the observation that mucoepidermoid carcinoma stem cells present high constitutive activity of the mTor pathway has considerable translational impact. Indeed, these results might lead to a new therapeutic target for this malignancy that will be explored in future studies by our laboratory. While we have concluded from our studies that ALDH^high^CD44^high^ cells demonstrate CSC properties, further research must be done to verify if both ALDH and CD44 play an active role in the maintenance of this stem cell phenotype. ALDH1 has been widely used as a cancer stem cells marker due to its role in normal stem cells function. However, whether or not it plays an active role in cancer stem cell maintenance in mucoepidermoid carcinoma is unclear. Further, little is known about the role of CD44 in the progression of mucoepidermoid carcinomas. CD44 has been shown to play an important role in resistance to radiation and chemotherapy and may play a role in tumor recurrence of head and neck squamous cell carcinomas [51]. The protein is encoded by one gene, but due to post-transcriptional modifications and alternative splicing, many variants of CD44 exist [51]. Studies have implicated CD44v6 to be more effective in isolating CSC, however, work in HNSCC showed similar levels of expression between CD44s and CD44v6 suggesting that this effect may be specific to various cancer types [52–56]. The antibody that was used in our studies was not specific to the CD44v6 splice variant. It is possible to using antibodies specific to this variant may lead to a further enrichment of the cancer stem cells in mucoepidermoid carcinomas, but this hypothesis was not tested here. Collectively, this work demonstrates that salivary gland mucoepidermoid carcinomas exhibit a small sub-population of cells with uniquely high tumorigenic potential. These cells can be identified by high ALDH activity and CD44 expression. Considering the role of cancer stem cells in tumor recurrence and resistance to therapy in other glandular cancers (*e.g.* breast, pancreatic), it is tempting to predict that these cells may also play a functional role in the relentless growth and resistance to therapy typically exhibited by human mucoepidermoid carcinomas. These results suggest that patients with mucoepidermoid carcinoma might benefit from the targeted ablation of this sub-population of uniquely tumorigenic cancer stem cells.

## MATERIALS AND METHODS

### Cell culture

Human salivary gland mucoepidermoid carcinoma cell lines (UM-HMC-1, UM-HMC-3A, UM-HMC-3B) previously characterized in our laboratory [38] were cultured in high glucose Dulbecco’s Modified Eagle’s Medium (DMEM; Invitrogen, Carlsbad, CA, USA) supplemented with penicillin/streptomycin (Invitrogen), L-glutamine (Invitrogen), 10% FBS (Invitrogen), 20 ng/ml epidermal growth factor (EGF; Sigma-Aldrich, St. Louis, MO, USA), 400 ng/ml hydrocortisone (Sigma-Aldrich), and 5 μg/ml insulin (Sigma-Aldrich) [38]. Cells were passaged using 0.05% trypsin/EDTA (Invitrogen). Primary human dermal microvascular endothelial cells (HDMEC; Lonza, Walkersville, MD, USA) were cultured using endothelial growth medium (EGM2-MV; Lonza).

### Flow cytometry

Trypsinized cells were filtered using 5 ml polystyrene round-bottom tumor with cell strainer caps (BD Pharmingen). Single cell suspensions of 2×10^6^ cells/ml were prepared and incubated with 5 μl Aldefluor^®^ substrate (BAA), or 5 μl of the inhibitor diethylaminobenzaldehyde (DEAB) for 40 minutes at 37°C, using the Aldefluor kit (StemCell; Vancouver, Canada). Cells were exposed to anti-CD44 (APC-Cat #559942, PE-Cat #550989), anti-CD24 (FITC-Cat #555427; BD Pharmingen), or anti-CD10 (APC-Cat #340923; BD Pharmingen) for 30 minutes at 4°C. Positive anti-HLA-ABC (PE-Cat #560168; BD Pharmingen) was used to separate human cells from mouse cells, and 7-AAD (Cat #00-6993-50; eBiosciences) staining was used to verify cell viability.

### Salisphere assay

Non-adherent spheres of salivary mucoepidermoid carcinoma cells (salispheres), previously characterized in normal salivary cells [57], were cultured in DMEM/F-12 (Invitrogen) supplemented with 20 ng/ml EGF (Sigma-Aldrich), 20 ng/ml basic fibroblast growth factor (bFGF; Millipore), 1% penicillin/streptomycin (Invitrogen), 1% glutamax (Invitrogen), 1% N-2 supplement (Invitrogen), 1 μM dexamethasone (Sigma-Aldrich), and 10 μg/ml insulin (Sigma-Aldrich) [39]. Cells were counted, diluted to 2,000 per 1.5 ml, and added to 6-well ultra-low attachment plates (Corning; Corning, NY, USA). For *in vitro* passaging, salispheres were collected and exposed to 0.25% trypsin for 5-10 minutes, and then mechanically dissociated. The trypsin was neutralized using a trypsin neutralizing solution (TNS; Lonza). Colonies of 50 cells or more were considered salispheres.

### *In vivo* studies

Single cell suspensions of sorted mucoepidermoid carcinoma cells (UM-HMC-1, UM-HMC-3A, UM-HMC-3B) were seeded in biodegradable scaffolds with 9 × 10^5^ human dermal microvascular endothelial cells (HDMEC; Lonza) and bilaterally implanted in the subcutaneous space on the dorsum of severe combined immunodeficient (SCID) mice (CB-17 SCID; Charles River, Wilmington, MA, USA), as we have shown [39, 40]. Second generation tumors were generated by transplanting cells retrieved from the digestion of the first generation tumors in secondary mice. Tumors were minced into small fragments and digested using 1X collagenase-hyaluronidase (Stem Cell Technologies; Vancouver, BC, Canada) at 37°C for 45 minutes, pipetting up and down every 15 minutes. Digested cells and tissues were passed through a 40-μm sieve (Fisher) and neutralized using 3-5 ml FBS. Cell suspensions were centrifuged and incubated with AKC lysis buffer (Invitrogen) for 1 minute, centrifuged, counted, and subjected to flow cytometry. For the studies designed to understand the effect of *in vitro* cell attachment conditions on the tumorigenic potential *in vivo*, cells were cultured with serum-free medium as salispheres (as described above), or in normal attachment conditions. After 7 days, attached cells were retrieved, 200,000 cells were seeded per biodegradable scaffold and transplanted into mice, as also described above. Alternatively, the salispheres were collected but not dissociated (to maintain the sphere structure), and 200,000 cells/scaffold were transplanted into mice. Tumor growth was measured every seven days with calipers, and mice were euthanized when the tumors reached a maximum of 2,000 mm^3^.

### Western blot

UM-HMC-3A and UM-HMC-3B were sorted for ALDH^high^CD44^high^. As controls, we combined the ALDH^high^CD44^low^, ALDH^low^CD44^high^, and ALDH^low^CD44^low^ as non-CSC cell population. NP-40 lysis buffer was used to prepare whole cell lysates that were resolved using PAGE. Membranes were probed using antibodies a 1:1000 dilution against human mTor, p-mTor, Akt, p-Akt, S6K, p-S6K (Cell Signaling; Beverly, MA, USA); 1:2000 dilution of EGFR, a 1:1000 dilution of p-EGFR, and beta-actin (Santa Cruz Biotechnology; Santa Cruz, CA, USA) overnight at 4°C.

## SUPPLEMENTARY MATERIAL FIGURES


