# Supplementary material for: ALDH/CD44 identifies uniquely tumorigenic cancer stem cells in salivary gland mucoepidermoid carcinomas
Source: Oncotarget. 2015 Sep 22;6(29):26633–50. doi: 10.18632/oncotarget.5782 (PMC4694941; doi:10.18632/oncotarget.5782)
Supplement: Supplementary file 1 [file oncotarget-06-26633-s001.pdf]

ALDH/CD44 identifies uniquely tumorigenic cancer stem cells in salivary gland mucoepidermoid carcinomas

Supplementary Material

Immunofluorescence Staining

Cells were cultured in Lab-Tek® 4-well chamber slides (Thermo Scientific) overnight, fixed with 2% formaldehyde and 0.2% glutaraldehyde for 20 minutes, incubated in 0.1% Triton-X 100 for 10 minutes, and then in 0.3% hydrogen peroxide for 10 minutes. Paraffin-embedded sections were deparaffinized and treated with a 1X antigen retrieval solution in citrate buffer (Dako; Carpinteria, CA, USA) at 45-98^{0}C for 60 minutes followed by treatment with 3% hydrogen peroxidase for 10 minutes. Rabbit anti-human ALDH1A1 (Abcam; Cambridge, England) was incubated overnight and labeled with Alexafluor 488 (Anti-Rabbit, Invitrogen) the following day. Cells were incubated with mouse anti-human CD44 (Thermo Scientific), rabbit anti-human CD10 (Abcam), and mouse anti-CD24 (Abcam) for 1 hour then labeled with Alexafluor 594 (anti-mouse, Invitrogen), Alexafluor 488 (anti-rabbit, Invitrogen), and Alexafluor 488 (anti-mouse, Jackson ImmunoResearch) respectively. We analyzed tissues from diagnostic incisional biopsies taken from 12 patients. A pathologist graded the samples according to WHO standards, but the tentative grades are subjective and observer-dependent.

Statistical analyses

Statistical analysis of *in vitro* data was performed using either a t-test or one-way ANOVA followed by post-hoc tests (SigmaStat 2.0 software; SPSS, Chicago, IL). Time to failure data was analyzed using the Kaplan-Meier method and the log-rank test. We employed linear mixed model regression to analyze the repeated measurements data on each tumor. Model fixed effects included time, marker, the interaction of time and marker, and model random effects included both mouse and tumor position. For all models a continuous autoregressive correlation structure was used, which assumes more correlated variances among temporally proximate observations. A log-transformation of the outcome variable (tumor volume) was to account for exponential increase of tumor volumes. Tumor growth rate since palpability (200 mm^3) was

measured, and we controlled for size differences of the tumor at first palpability. Analysis was performed using the “nlme” package in the statistical software program R v 3.1.0. (41).

A

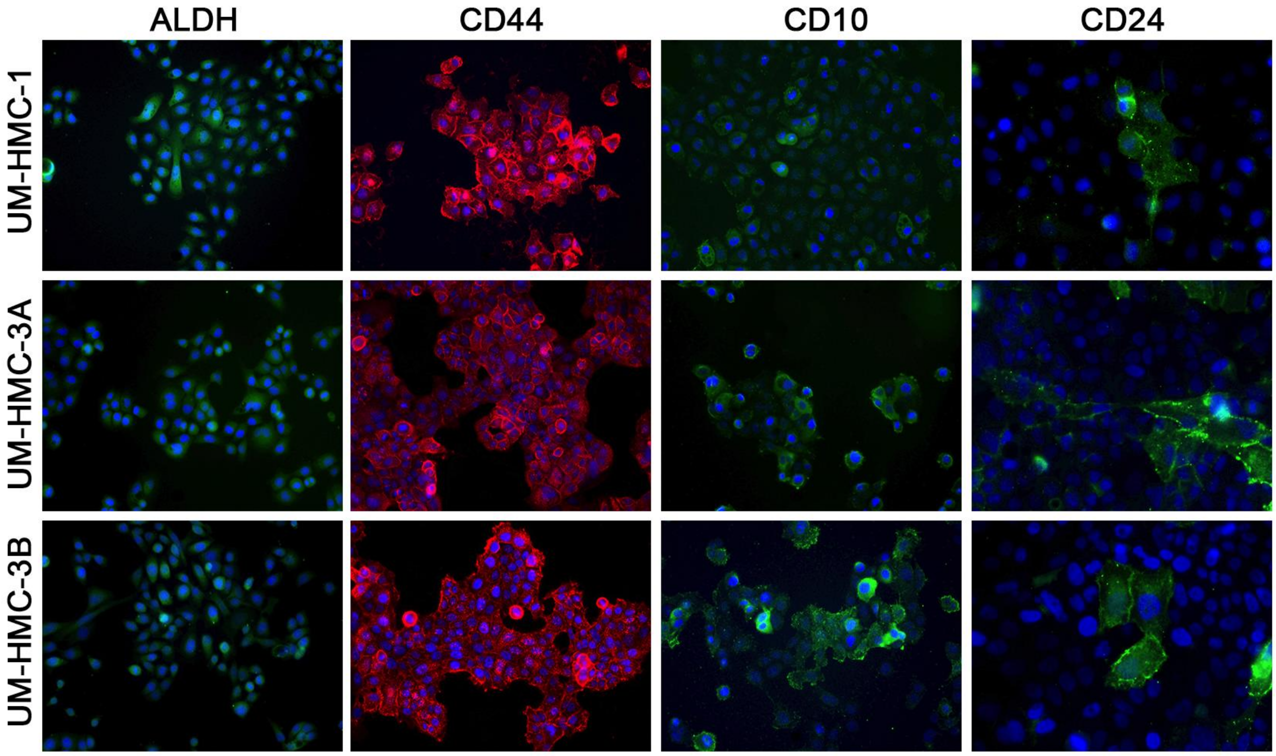

Supplementary Figure S1. Characterization of putative stem cell markers in human mucoepidermoid carcinoma cell lines. A, Representative photomicrographs of immunofluorescence staining for ALDH, CD44, CD10, or CD24 of a panel of mucoepidermoid carcinoma cells (UM-HMC-1, UM-HMC-3A, UM-HMC-3B) cultured in Lab-Tek chamber slides. Images were taken at 200X.

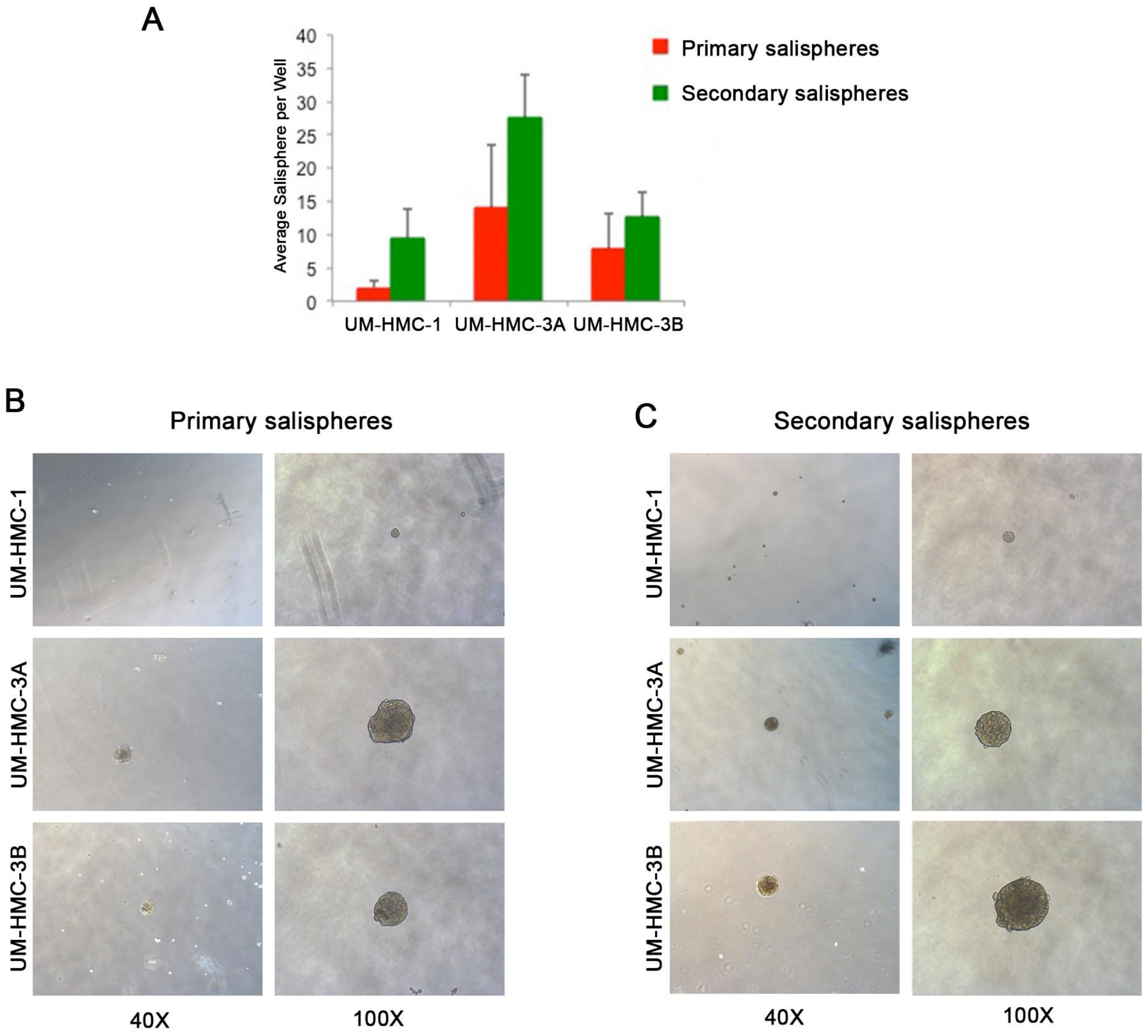

Supplementary Figure S2. Sphere analysis of unsorted HMC cells. (A) Graph depicting the average number of sphere formed per well in a 6-well low attachment plate in UM-HMC-1, UM-HMC-3A, and UM-HMC-3B cells. (B) Images of UM-HMC-1, UM-HMC-3A, and UM-HMC-3B primary spheres in low attachment culture. Images were taken at 40X and 100X. (C) Images of UM-HMC-1, UM-HMC-3A, and UM-HMC-3B secondary spheres in low attachment culture. Images were taken at 40X and 100X.

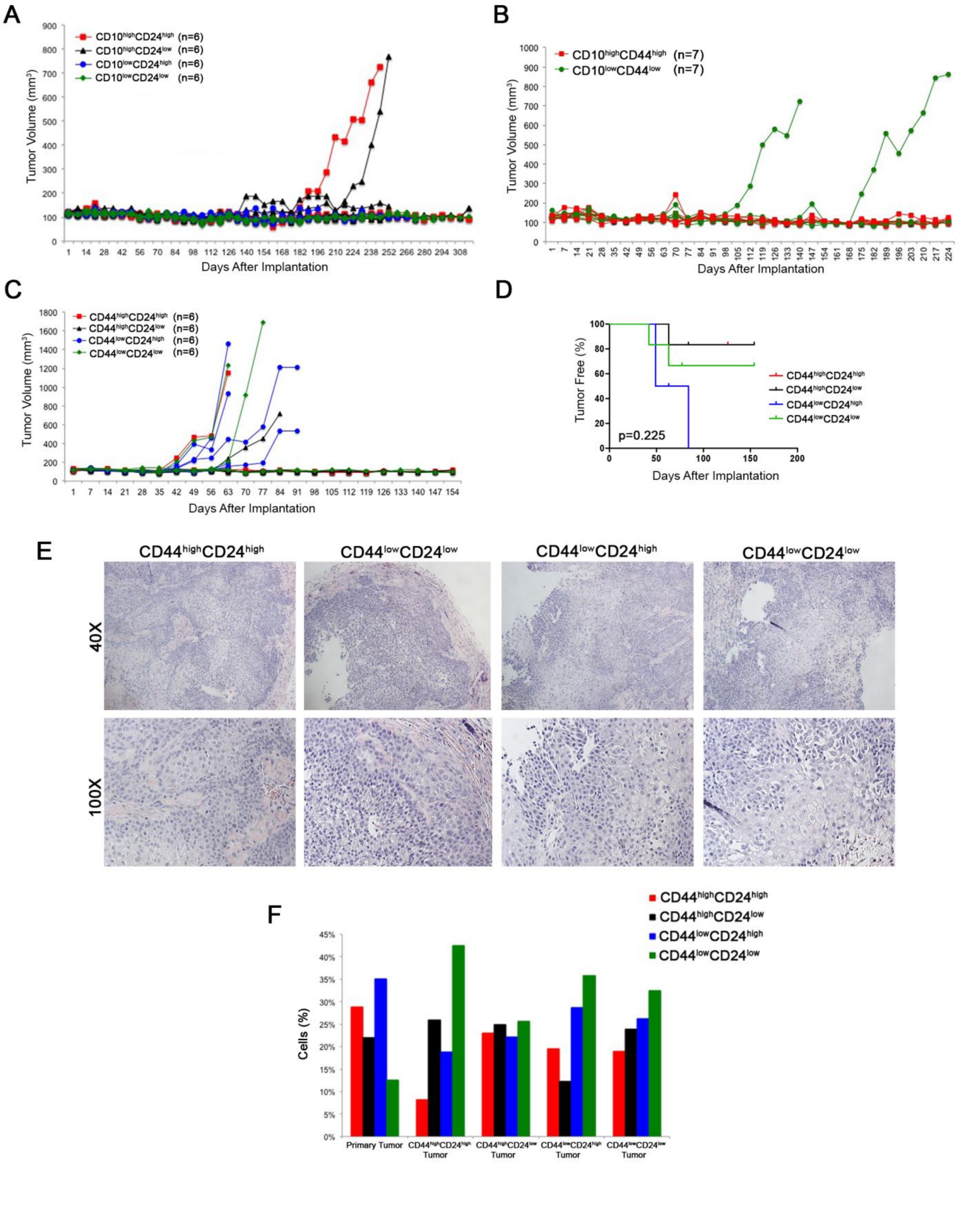
A
Tumor Volume (mm3)
Days After Implantation
CD10highCD24high (n=6)
CD10highCD24low (n=6)
CD10lowCD24high (n=6)
CD10lowCD24low (n=6)
B
CD10highCD44high (n=7)
CD10lowCD44low (n=7)
C
CD44highCD24high (n=6)
CD44highCD24low (n=6)
CD44lowCD24high (n=6)
CD44lowCD24low (n=6)
D
Tumor Free (%)
p=0.225
CD44highCD24high
CD44highCD24low
CD44lowCD24high
CD44lowCD24low
E
CD44highCD24high
CD44lowCD24low
CD44lowCD24high
CD44lowCD24low
40X
100X
F
Cells (%)
Primary Tumor
CD44highCD24high Tumor
CD44highCD24low Tumor
CD44lowCD24high Tumor
CD44lowCD24low Tumor

Supplementary Figure S3. *In-Vivo* tumorigenicity of low passage CD44/CD24 sorted cells. (A) Tumor growth chart of CD10/CD24 sorted cells. Scaffolds were seated with 2,000 CD10/CD24 sorted cells and 900,000 HDMEC cells. (B) Tumor growth chart of CD10/CD44 sorted cells. Scaffolds were seated with either 500 $CD10^{high}CD44^{high}$ cells or 5,000 $CD10^{low}CD44^{low}$ cells along with 900,000 HDMEC cells. (C) Tumor growth chart of tumors generated from UM-HMC-3B (passage 27) 9th generation xenograft cells that were sorted according to the expression of CD44/CD24. (D) Graph depicting the time to palpability of CD44/CD24 sorted tumors. Tumors were considered palpable once they reached 200 mm^3. (E) H&E staining of sections taken from UM-HMC-3B (passage 27) 9th generation xenograft CD44/CD24 sorted cells. (F) Flow cytometry analysis of UM-HMC-3B (passage 27) 9th generation xenograft CD44/CD24 sorted tumors. Primary tumors were digested and stained for CD44 and CD24.
